# Abscisic acid negatively interferes with basal defence of barley against *Magnaporthe oryzae*

**DOI:** 10.1186/s12870-014-0409-x

**Published:** 2015-01-21

**Authors:** Sylvia Ulferts, Rhoda Delventhal, Richard Splivallo, Petr Karlovsky, Ulrich Schaffrath

**Affiliations:** Department of Plant Physiology, RWTH Aachen University, 52056 Aachen, Germany; Institute for Molecular Biosciences, Goethe University of Frankfurt, 60438 Frankfurt am Main, Germany; Molecular Phytopathology and Mycotoxin Research, University of Goettingen, Grisebachstrasse 6, 37077 Goettingen, Germany

**Keywords:** Penetration resistance, Rice blast, Head blast, Quantitative microscopy, Biotic stress

## Abstract

**Background:**

Plant hormones are well known regulators which balance plant responses to abiotic and biotic stresses. We investigated the role of abscisic acid (ABA) in resistance of barley (*Hordeum vulgare* L.) against the plant pathogenic fungus *Magnaporthe oryzae*.

**Results:**

Exogenous application of ABA prior to inoculation with *M. oryzae* led to more disease symptoms on barley leaves. This result contrasted the finding that ABA application enhances resistance of barley against the powdery mildew fungus. Microscopic analysis identified diminished penetration resistance as cause for enhanced susceptibility. Consistently, the barley mutant *Az34*, impaired in ABA biosynthesis, was less susceptible to infection by *M. oryzae* and displayed elevated penetration resistance as compared to the isogenic wild type cultivar Steptoe. Chemical complementation of *Az34* mutant plants by exogenous application of ABA re-established disease severity to the wild type level. The role of ABA in susceptibility of barley against *M. oryzae* was corroborated by showing that ABA application led to increased disease severity in all barley cultivars under investigation except for the most susceptible cultivar Pallas. Interestingly, endogenous ABA concentrations did not significantly change after infection of barley with *M. oryzae*.

**Conclusion:**

Our results revealed that elevated ABA levels led to a higher disease severity on barley leaves to *M. oryzae*. This supports earlier reports on the role of ABA in enhancing susceptibility of rice to the same pathogen and thereby demonstrates a host plant-independent function of this phytohormone in pathogenicity of monocotyledonous plants against *M. oryzae*.

**Electronic supplementary material:**

The online version of this article (doi:10.1186/s12870-014-0409-x) contains supplementary material, which is available to authorized users.

## Background

Generally, plant hormones are small molecules derived from different metabolic pathways that act at low concentrations either locally or distantly from the site of synthesis [1]. Apart from being important for development, plants use their hormone network to respond to external stimuli such as abiotic and biotic stresses. Salicylic acid (SA), jasmonic acid (JA) and ethylene (ET), the so-called immunity hormones [2], are best known because of their major function in regulating disease resistance in many plant species against a plethora of pathogens. Importantly, they do not act independently from each other but rather form a multidimensional network with synergistic or antagonistic interactions in response to pathogens with different life-styles [3]. More precisely, the ability of a plant to resist a pathogen depends on hormonal balance rather than on the absolute concentration of individual hormones [4]. Pathogens target this sensitive equilibrium to their advantage for promoting disease. Thus, they either produce plant hormones themselves, like e.g. *Agrobacterium tumefaciens* (indole-3-acetic acid) or *Giberella fujikuroi* (gibberellic acid) [4], or synthesize hormone-like substances such as coronatine, a JA-mimic secreted by *Pseudomonas syringae* [5]. For Arabidopsis it was shown that distinct hormone pathways are effective only against subsets of pathogens, e.g. SA-dependent resistance acts most efficiently against biotrophic pathogens which solely colonize living plant tissue. It was shown that some biotrophs developed the ability to suppress SA-mediated defence by up-regulating the antagonistical JA/ET pathway [3].

The classical plant hormone abscisic acid (ABA) also antagonises SA-mediated defence as shown e.g. in *Arabidopsis,* where ABA-treatment increased the susceptibility to an avirulent strain of *Pseudomonas syringae* pv. *tomato* by suppressing lignin accumulation and defence gene expression [6]. Also for monocotyledonous plants such as rice a negative correlation in resistance against *Xanthomonas oryzae* pv. *oryzae* of ABA- and SA-signalling was reported [2]. In turgid plants, ABA-biosynthesis takes place in vascular bundles. Plant ABA is a terpenoid with 15 carbon atoms derived from C_40_ carotenoids that are produced via the 2-C-methyl-d-erythritol-4-phosphate (MEP) pathway [7]. By contrast fungal ABA, produced e.g. by *Cercospora spp.* or *Botrytis cinerea*, is derived from the MVA (mevalonic acid) pathway [8]. This difference in biosynthetic pathways suggests independent acquisition of ABA-metabolism in fungi and plants. ABA can affect the outcome of plant disease either negatively, most likely due to its interference with SA-signalling, or positively, e.g. by its involvement in primed callose deposition [9,10].

**Figure 1:**
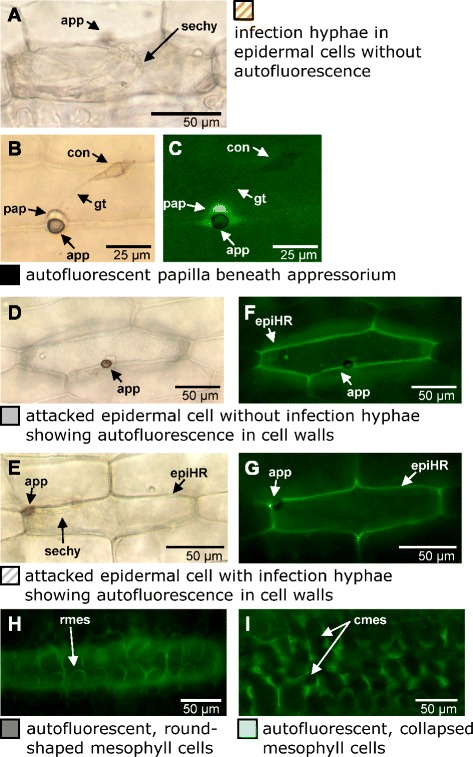
**Microscopic evaluation of the infection of** ***M. oryzae*** **on barley.** Primary leaves of barley cultivar Ingrid were inoculated with a spore solution of *M. oryzae* isolate TH6772 (200,000 conidia mL^−1^) seven days after sowing. Leaves were harvested at 72 h p.i. and placed in 25% acetic acid in ethanol (v/v) until bleached. Thereafter, leaves were analyzed in water by bright field **(A, B, D and E)** or epi-fluorescence **(C, F, G, H and I)** microscopy. Category designations and labels correspond to the quantitative evaluation in Figures 2, 3 and 5C. app: appressorium; sechy: secondary hyphae; con: conidium; pap: papilla; gt: germ tube; epiHR: epidermal hypersensitive response; rmes: round-shaped mesophyll cells; cmes: collapsed mesophyll cells.

For hemi-biotrophic pathogens, such as *Magnaporthe oryzae*, less is known about the function of ABA in plant resistance. *M. oryzae* is a major fungal pathogen of rice (*Oryza sativa* L.) but is also able to infect other grasses or sedges including barley and wheat [11-13]. Koga and co-workers [14] found that ABA-treatment suppressed resistance of rice plants against *M. oryzae*. Interestingly, Wiese et al. [15] reported the opposite effect for the barley/powdery mildew (*Blumeria graminis* f. sp. *hordei*, *Bgh*) interaction. *M. oryzae* invades barley plants by direct penetration of epidermal cells which takes place after germination of conidiospores and formation of dark-pigmented appressoria. Growth of invasive hyphae into epidermal cells can happen without microscopically visible plant reaction (Figure 1A). However, also an autofluorescent papilla, a fortification formed at the inner site of the epidermal cell wall, may occur beneath appressoria (Figure 1B, C). Additional cytological reactions of barley cells attacked by *M. oryzae* are autofluorescent walls of epidermal cells (Figure 1D-G) or round-shaped and collapsed mesophyll cells, respectively (Figure 1H, I). The initial infection process, up to the formation of bulbous infection hyphae in the primarily attacked epidermal cell, resembles a biotrophic interaction. Later stages of infection, by contrast, are associated with cell necrosis which is visible at the cellular level as collapsed autofluorescent mesophyll tissue (Figure 1I) [16,17].

We have investigated the interaction between barley and *M. oryzae* for about 15 years, elucidating e.g. different aspects of quantitative or nonhost resistance [17-20]. A so far unexplored aspect was the function of plant hormones in this interaction. In the present study we closed this gap by identifying ABA as a balancing factor which contributes to susceptibility. Consequently, a barley mutant with a defect in ABA biosynthesis exerted enhanced resistance to *M. oryzae*. Quantification of endogenous ABA revealed differences among cultivars but no substantial changes during infection with *M. oryzae*.

## Results

### ABA-treatment increased susceptibility of barley against *M. oryzae*

**Figure 2:**
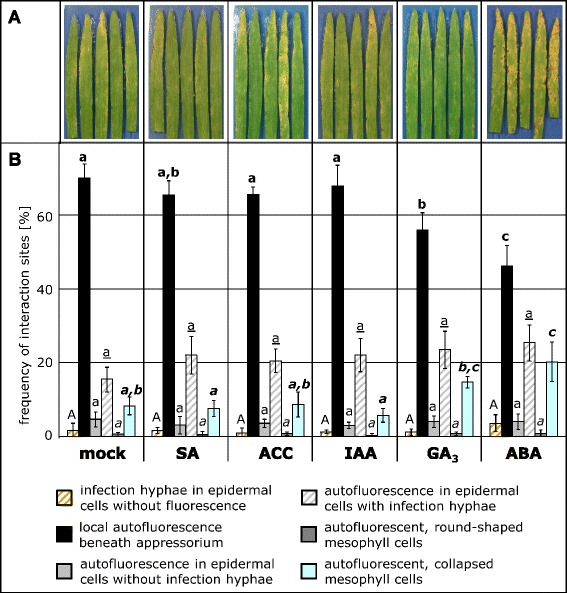
**Effect of phytohormone application on the infection of barley with** ***M. oryzae*** **.** Primary leaves of barley cultivar Ingrid were sprayed with the following solutions seven days after sawing: sodium salicylate (SA, 0.1 mM), 1-aminocyclopropane-1-carboxylic acid (ACC, 20 μM), indole-3-acetic acid (IAA, 20 μM), gibberellic acid (GA_3_, 20 μM), abscisic acid (ABA, 20 μM) or mock solution, respectively. One hour after treatment the plants were inoculated with conidia of *M. oryzae* isolate TH6772 (200,000 conidia mL^−1^). Representative leaves of each treatment seven days after inoculation are depicted in **(A)**. Individual plant-fungus interaction sites were inspected microscopically on leaves harvested at 72 h p.i. **(B)** and assigned to categories as depicted in Figure 1. Bars represent means and standard deviations of four leaves with at least 100 interaction sites evaluated per leaf. Significant differences were determined for each category using One Way ANOVA (p ≤ 0.05) and marked by different letters. The experiment was repeated once with a similar result.

In a first exploratory experiment, we investigated which of the classical plant hormones influences the interaction between barley and *M. oryzae*. Therefore, primary leaves of barley were sprayed with test-solutions of salicylic acid (SA), abscisic acid (ABA), gibberellic acid (GA_3_), auxin (IAA) and the ethylene precursor 1-aminocyclopropane-1-carboxylic acid (ACC) and inoculated after one hour with the pathogen. Typical disease symptoms developed on mock-treated control plants as spindle-shaped lesions indicating that the fungus had successfully completed its life-cycle and produced conidia (Figure 2A). Hormone- and mock-treated plants were compared macroscopically after seven days and no substantial differences in disease severity were found for most of the hormone treatments (Figure 2A). The treatment with ABA, however, led to more frequent and larger disease symptoms on treated leaves. To exclude the possibility of a direct effect of ABA against the pathogen, an additional experiment was performed in which solutions with different ABA-concentrations were applied by soil drench and inoculation with *M. oryzae* was done after 48 hours (Additional file 1: Figure S1). Quantitative measurement of disease symptoms after seven days revealed that each concentration of ABA significantly increased disease severity. Thus, a treatment with 20 μM ABA doubled the number of lesions, whereas a treatment with 100, 200 or 300 μM caused a three to four time increase.

We followed this observation in more depth by quantitative microscopic analysis of the infection process using a combination of bright-field and epi-fluorescence microscopy. Generally, barley can arrest or hinder disease progress of *M. oryzae* at penetration or post-penetration stages, both of which can be tracked by monitoring the presence or absence of invasive hyphae in attacked epidermal cells and its coincidence with the occurrence of autofluorescent plant material. Accordingly six categories of disease progress were discriminated as depicted in Figure 1. For quantitative assessment, microscopic samples were harvested at 72 h p.i. and individual infection sites were assigned to one of these categories. At most plant-fungus interaction sites (approx. 65-70%) a local deposition of autofluorescent material was observed beneath fungal appressoria and in association with a papilla (Figure 2B). This was the case for mock-treated, SA, ACC and IAA-treated plants. For this group of plants, autofluorescence of collapsed mesophyll cells, indicating accelerated proliferation of the pathogen, was found at only 5-10% of infection sites. By contrast, the latter category was by trend more frequent (17%) in GA_3_-treated and even more and significantly frequent (20%) in ABA-treated plants (Figure 2B). Concomitant with the increase in mesophyll colonisation a significantly decreased number of infection sites were found which were assigned to the category “local autofluorescence beneath appressorium”, suggesting a more efficient growth of the pathogen from attacked epidermal cells into the underlying mesophyll.

**Figure 3:**
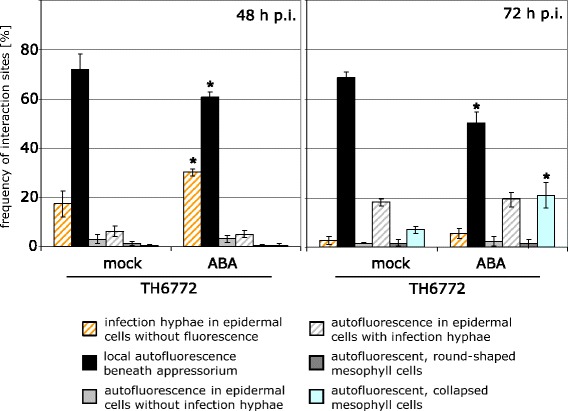
**Disease progression of** ***M. oryzae*** **on ABA-treated barley plants.** Seven day old primary leaves of barley cultivar Ingrid were sprayed either with abscisic acid solution (20 μM) or mock-solution and inoculated one hour later with *M. oryzae* isolate TH6772 (200,000 conidia mL^−1^). At 48 and 72 h p.i. leaves were harvested for microscopic analysis and plant-fungus interaction sites were assigned to categories shown in Figure 1. Bars represent mean and standard deviation of four leaves with at least 100 interaction sites analysed per leaf. Significant differences as determined by t-test (p ≤ 0.05) are marked with asterisks. The experiment was repeated twice with similar results.

Further experiments focused on ABA and its interference with the resistance of barley against *M. oryzae*; our observations with GA_3_ will be followed up elsewhere. Since our results pointed to a potential function of ABA in the initial infection process, we evaluated early penetration events of *M. oryzae* on barley leaves harvested at different time points after inoculation. Again, individual infection sites were inspected in a quantitative manner and assigned to the categories described above. At 48 h p.i. the number of infection sites grouped into the category “local autofluorescence beneath appressorium” was significantly less in ABA-treated (60%) as compared to mock-treated plants (70%) (Figure 3). This phenomenon was accompanied by more infection sites in the category “infection hyphae in epidermal cell without autofluorescense” for ABA-treated plants. Taken together, these results may be interpreted as if the ABA-treatment negatively interferes with early pathogen recognition by the plant. An alternative interpretation could be that the ABA-treatment directly influenced biosynthesis or accumulation of autofluorescent material. Strikingly, the frequency of interaction sites assigned to category “infection hyphae in epidermal cell without fluorescence” declined dramatically from 48 to 72 h p.i. for ABA-treated plants whereas the frequency of interaction sites found for the category “autofluorescence, collapsed mesophyll cells” increased at the same magnitude (Figure 3). This indicates a correlation of diminished autofluorescent response in attacked epidermal cells with accelerated pathogen spreading into the mesophyll and is in accordance with observations previously reported by Zellerhoff et al. [13,21]. Concomitantly, the frequency of interaction sites assigned to the category “local autofluorescence beneath appressorium” was almost equal between 48 and 72 h p.i. for ABA-treated plants (Figure 3), suggesting that at these sites fungal infection was aborted.

**Figure 4:**
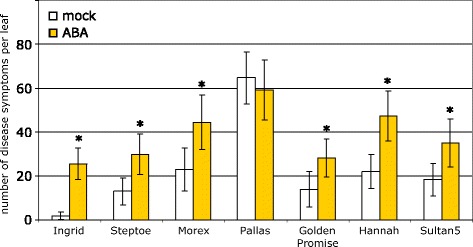
**Cultivar-specific differences in disease severity after ABA-treatment.** Abscisic acid (20 μM) or mock-solution were sprayed onto seven-days-old primary leaves of barley cultivars Ingrid, Steptoe, Morex, Pallas, Golden Promise, Hannah and Sultan5. Inoculation with *M. oryzae* isolate TH6772 (200,000 conidia mL^−1^) took place one hour after treatment. Disease severity was evaluated six days after inoculation by counting blast lesions. Means and standard deviations of at least nine leaves per cultivar and treatment are shown. Significant differences between mock- and ABA-treatment were determined individually for each cultivar using t-test (p ≤ 0.05) and marked with an asterisk. The experiment was repeated twice with similar results.

So far, all experiments on the influence of ABA in the pathosystem barley/*M. oryzae* were done solely with the cultivar Ingrid. It could not be excluded, therefore, that the observed response to ABA was a specific feature of this particular cultivar. To address this question, we extended the study to seven barley cultivars encompassing spring and winter varieties. All plants were sprayed with a 20 μM ABA solution seven days after sowing and inoculated with *M. oryzae* isolate TH6772. Disease symptoms developed on leaves of all cultivars, indicating a compatible interaction with the chosen pathogen isolate (Figure 4). Quantitative differences in the number of lesions per leaf were found on mock-treated plants which revealed that the cultivars exhibited different levels of basal resistance against *M. oryzae*. Thus, on cv. Ingrid on average only two to five lesions were found per leaf of mock-treated plants. The number of disease symptoms per leaf increased for ABA-treated Ingrid-plants to 26, which was the highest relative rise within this experiment (Figure 4). For Steptoe, Morex, Golden Promise, Hannah and Sultan5 the number of lesions on ABA-treated plants was twice as high as on mock-treated plants of the same cultivar (Figure 4). The cultivar Pallas was an exception in this regard, since the overall number of lesions on untreated plants was highest (62 lesions per leaf) and ABA-treatment did not further increase disease severity. The disproportionately higher numbers of lesions on cv. Pallas may indicate a compromised basal defence of this cultivar against *M. oryzae* isolate TH6772. In case this impairment affects a resistance pathway that is influenced by ABA, additional ABA would not lead to a further decline in resistance.

### Reduced ABA-content enhanced resistance of barley against *M. oryzae*

**Figure 5:**
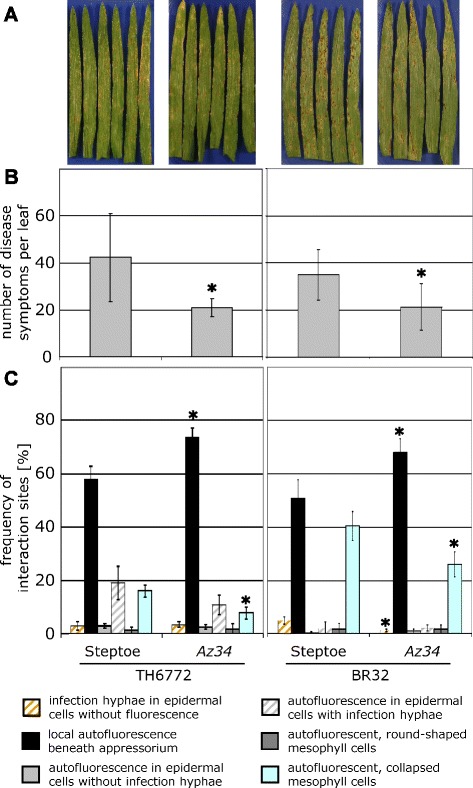
**Infection phenotype of** ***M. oryzae*** **on barley mutant** ***Az34*** **which is impaired in ABA-biosynthesis.** Disease symptoms on primary leaves of barley cultivar Steptoe and the mutant *Az34*, inoculated either with *M. oryzae* isolate TH6772 (200,000 conidia mL^−1^) or isolate BR32 (100,000 conidia mL^−1^), are depicted at seven and six days after inoculation, respectively **(A)**. Blast lesions were counted for each genotype and treatment. Means and standard deviations calculated for eight leaves are shown **(B)**. Leaves harvested at 72 h p.i. were analysed by microscopy **(C)**. Categorisation of plant-fungus interaction sites was done according to Figure 1. Bars represent means and standard deviations for four leaves with at least 100 interaction sites inspected per leaf. Significant differences between Steptoe and mutant plants observed in **(B)** and **(C)** were determined using t-test (p ≤ 0.05) and marked with asterisks. For **(C)** the significance was tested separately for each category. The experiment was repeated twice with similar results.

Hitherto, our results accounted for a regulatory function of ABA in resistance of barley against *M. oryzae*. To further validate this finding in an independent experimental set-up, we evaluated whether reduced levels of ABA would lead to the opposite effect, i.e. an increase in resistance of barley to this pathogen. Therefore, we made use of the existing barley mutant *Az34* which is impaired in the ability to produce ABA due to a mutation in a gene controlling a molybdenum cofactor [22]. This mutation results in deficiency in molybdoenzymes such as aldehyde oxidase which e.g. has ABA aldehyde, a putative ABA precursor, as substrate [22]. *Az34* was generated in the genetic background of cultivar Steptoe for which we already had shown that it is susceptible to *M. oryzae* isolate TH6772 and that exogenous application of ABA increased the number of lesions (Figure 4). Macroscopic comparison of inoculated leaves from Steptoe wild type plants with the *Az34* mutant indicated a slightly lower disease severity on mutant leaves (Figure 5A) which was quantitatively confirmed by lesion counting (Figure 5B). A macroscopically clearer result was obtained by inoculation of both genotypes with *M. oryzae* isolate BR32 which caused larger lesions on infected leaves (Figure 5A). Even in this case a significant reduction in the number of lesions was observed on mutant leaves (Figure 5B).

Additionally, a microscopic analysis of cellular defence reactions was performed, using the classification scheme described above (Figures 2 and 3). Significant differences between Steptoe and *Az34* mutant plants were observed for the categories “local autofluorescence beneath appressorium” and “autofluorescent, collapsed mesophyll cells” (Figure 5C). The frequency of interaction sites grouped in the first category was higher in the mutant as compared to Steptoe while the frequency of interaction sites assigned to the latter category was lower, indicating more efficient block of penetration and less effective invasion of the pathogen into the mesophyll of mutant plants. This result was found with both *M. oryzae* isolates TH6772 and BR32, underpinning the general validity of the observation.

**Figure 6:**
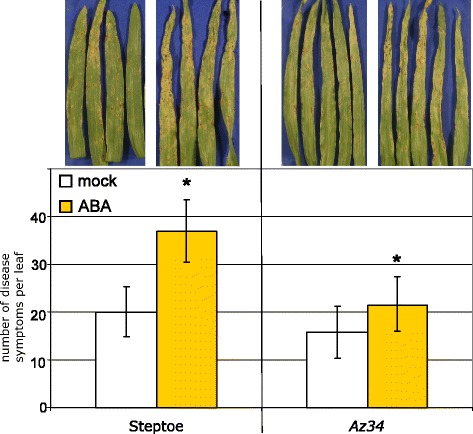
**Chemical complementation of** ***Az34*** **mutant phenotype by ABA-treatment.** Leaves of seven-day-old barley plants from cultivar Steptoe or mutant *Az34* were sprayed either with abscisic acid (20 μM) or mock solution. Inoculation was done one hour later with *M. oryzae* isolate TH6772 at a spore density of 200,000 conidia mL^−1^. Pictures were taken seven days post inoculation. Quantification of disease severity was done by counting blast lesions. Bars represent means and standard deviations of ten leaves and significant differences (t-test, p ≤ 0.05) are indicated with an asterisk. The experiment was repeated once with a similar result.

**Figure 7:**
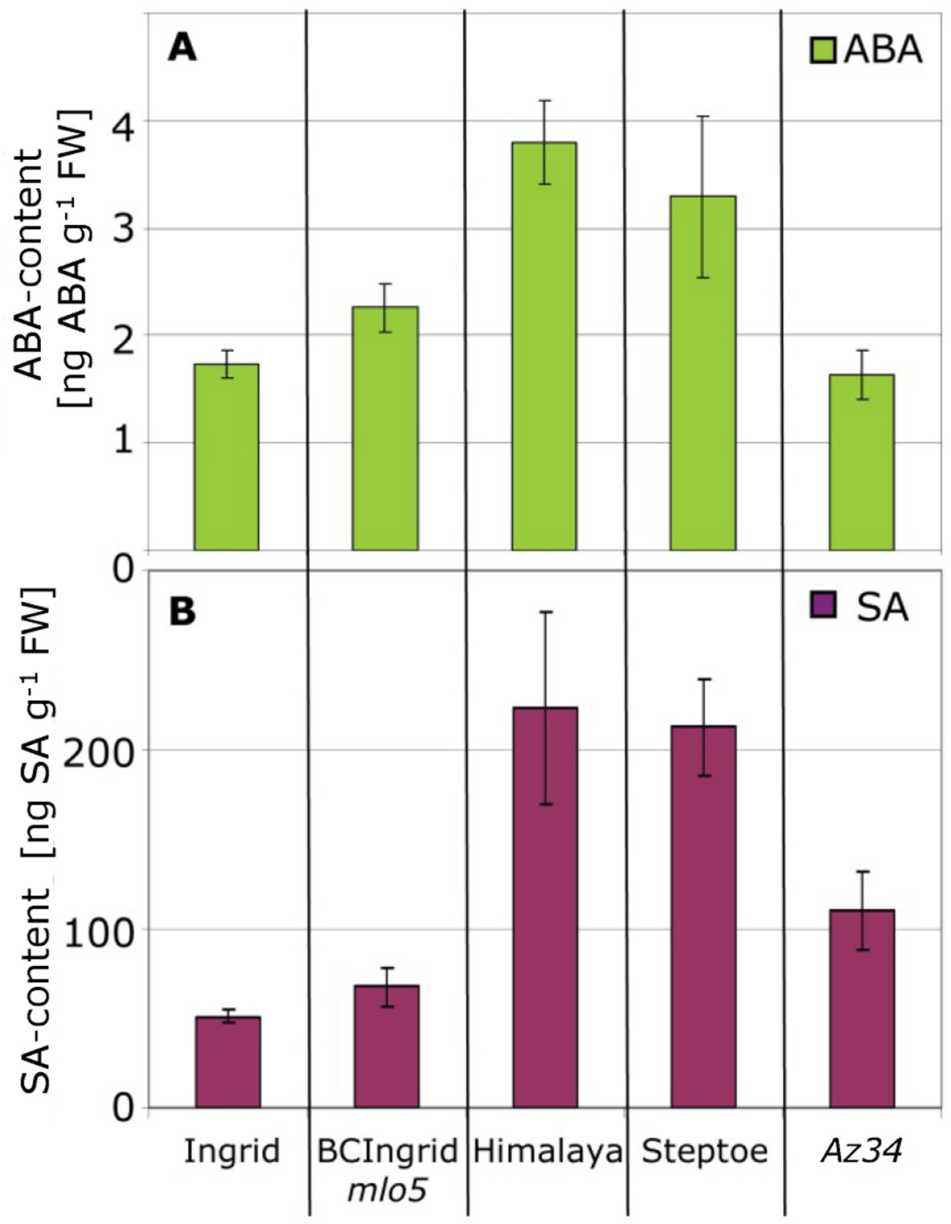
**Abscisic acid and salicylic acid level in different barley genotypes.** Seven-day-old primary barley leaves were harvested from cultivar Ingrid, Himalaya, Steptoe, the backcross line BCIngrid*mlo5*, and the mutant *Az34*, respectively. Samples consisting of five leaves were analysed by HPLC-MS-MS for ABA **(A)** or SA **(B)** content. Means and standard deviations for three samples harvested in a single experiment are shown. The experiment was repeated twice for ABA and once for SA determination with similar results.

We verified our finding that the reduced ABA-content in *Az34* mutant plants was the cause for a lower degree of susceptibility against *M. oryzae* by chemical complementation. Therefore mutant plants were sprayed with a 20 μM solution of ABA prior to inoculation. Indeed, exogenous application of ABA slightly but significantly increased the number of lesions on *Az34* mutant plants to a level as observed on wild type plants (Figure 6). Interestingly, the number of disease symptoms on chemically complemented *Az34* mutant plants was still lower than observed for ABA-treated Steptoe wild type plants. Endogenous ABA-levels were 3.2 ng per g fresh weight for the cultivar Steptoe and approximately half of that for the *Az34* mutant (Figure 7), indicating that ABA-biosynthesis was compromised rather than completely abolished in the mutant.

### Endogenous ABA-levels correlated with susceptibility of cultivars but were not affected by infection

**Figure 8:**
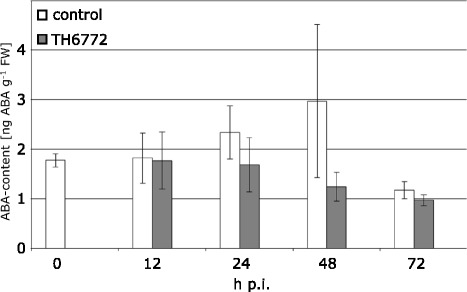
**Kinetic of ABA content in barley after inoculation with** ***M. oryzae*** **.** Primary leaves of barley plants (cultivar Ingrid) inoculated either with *M. oryzae* isolate TH6772 (200,000 conidia mL^−1^) or a mock solution without spores were harvested at time points indicated and subjected to HPLC-MS-MS analysis. Bars shown are means and standard deviations of measurements for seven samples harvested in three independent experiments (one experiment with harvest of a single and two experiments with harvest of duplicate samples). No significant differences between inoculated and mock-treated samples were found using One Way ANOVA (p ≤ 0.05).

Our experiments revealed a higher susceptibility of barley cv. Steptoe to infection with *M. oryzae* as compared to cv. Ingrid (Figure 4) and an increase in susceptibility to infection in both cultivars after ABA treatment. Endogenous ABA level in Steptoe was twice as high as in Ingrid (Figure 7A), corroborating the role of ABA in susceptibility. ABA analysis in further cultivars and in the mutant *Az34* showed that the ABA level in Steptoe was not unique and that the mutant *Az34* contained a comparable level of ABA to cv. Ingrid (Figure 7A). Because ABA is known to suppress the SA-dependent defence pathway [2,9,23,24] as well as SA-mediated induction of systemic acquired resistance [25], we determined the content of free SA in leaf extracts of the same cultivars as used for ABA analysis. Interestingly, SA and ABA concentrations were correlated in the different barley genotypes (Figure 7B) as indicated by a correlation coefficient of 0.8967 (p-value 0.039, both calculated by Pearson Product Moment Correlation using SigmaStat). To elucidate changes in endogenous levels of ABA after inoculation with *M. oryzae*, leaf samples of inoculated and mock-treated cv. Ingrid plants were harvested in a time course and subjected to HPLC-MS analysis. Although some variation in ABA-content did occur during the observation period, no significant differences were found between mock-treated and inoculated plants (Figure 8).

## Discussion

The phytohormone ABA is best known to be involved in seed dormancy and senescence. Also a function of ABA in controlling stomatal aperture and in plant responses to environmental changes such as water deficiency was demonstrated [26]. More recently a new facet was added to this picture by showing that ABA is a modulator of plant pathogen interactions. Depending on the pathosystem under investigation, the ABA-effect can range from promoting disease to increase resistance [9,27]. Our results show that exogenous application of ABA to barley compromises resistance against *M. oryzae* in a quantitative manner (Figure 2A). We investigated the ABA-mediated increase in susceptibility in detail by a quantitative cytological assessment of early infection stages of *M. oryzae*. In ABA-treated plants fungal infection sites were significantly less frequently grouped into the category “local autofluorescence beneath appressorium” at 72 h p.i.; instead, the fungus was more frequently able to cause cell collapse in the mesophyll (Figure 2B). This result suggested an enhanced penetration success and a more rapid transition of *M. oryzae* from the epidermis into the mesophyll. A similar effect on diminishing the basal defence of barley against *M. oryzae* was reported for the application of Cytochalasin E, an inhibitor affecting the actin cytoskeleton [17]. Further evidence that ABA supports the invasion of *M. oryzae* into barley leaves was provided by a comparison of the infection progress at different time points after inoculation. At 48 h p.i. *M. oryzae* infection hyphae that did not cause accumulation of autofluorescent material in epidermal cells were found more often after ABA treatment than in untreated controls (Figure 3). This can be accounted for interference of ABA with the recognition of the fungus by its host, facilitating unnoticed penetration, or by direct inhibition of a biochemical process that leads to the accumulation of autofluorescent material. The latter hypothesis is corroborated by a report that ABA down-regulated phenylalanine ammonia-lyase, an enzyme generating autofluorescing phenolic compounds, e.g. in soybean [28]. The effect of ABA on the recognition of a pathogen by its host is also conceivable because ABA-treatment increased the resistance of barley against powdery mildew and enhanced the susceptibility of rice plants to *M. oryzae* [14,15]. A further example for such ambivalence was shown for the inverse effectiveness of the *mlo* resistance allele against these pathogens [19]. This phenomenon may be explained by different life-styles, biotrophy versus hemi-biotrophy, of these pathogens. Noteworthy, our results are in accordance with the work published by Koga *et al.* [14], indicating a host plant-independent mechanism by which ABA enhances susceptibility to *M. oryzae*. Koga and co-workers found that increased *de novo* synthesis of ABA under low temperature conditions is responsible for rendering rice plants more susceptible to *M. oryzae*. This finding was supported by our results with barley mutant *Az34*, which is impaired in *de novo* biosynthesis of ABA after water stress [22], and which we found to be more resistant to *M. oryzae* (Figure 5). The role of ABA in these phenomena was confirmed by chemical complementation: application of ABA onto leaves of mutant plants re-established higher susceptibility against the pathogen (Figure 6).

We could not detect an increase in ABA-levels in barley leaves infected with *M. oryzae* (Figure 8), presumably because extraction of whole leaves masked effects occurring locally at infection sites. The effect of fungal infection on the ABA level in a host is known to vary even for the same pathogen. For instance, no increase of ABA in xylem of *B. napus* colonized with *V. longisporum* was observed [29], though infection of *A. thaliana* with the same fungus dramatically induced ABA levels in the shoot [30]. Jiang et al. [23] detected ABA in hyphae, conidia and culture media of *M. oryzae*, suggesting that the fungus might secrete this plant hormone to actively suppress plant defence. In this scenario, ABA most likely acts via its antagonistic interaction against SA- and ethylene-dependent signalling pathways in the resistance of rice against *M. oryzae* [23,31]. We have not found any negative correlation between basal levels of ABA and SA in barley. Performing Northern blot analysis, we also have not found a down-regulation of the SA marker gene *PR1b* after ABA-treatment in barley plants inoculated with *M. oryzae* (Additional file 1: Figure S2). Together this might indicate that suppression of the SA pathway alone might not be responsible for the ABA-mediated enhancement of susceptibility of barley to *M. oryzae*.

Quantitative cytological assessment revealed that application of GA_3_, similar to ABA, led to a lower number of infections sites at which autofluorescence occurred beneath appressoria (Figure 2), a cellular reaction clearly associated with diminished penetration resistance [17]. This observation is in accordance with the results published by Yang and co-workes [32] who demonstrated that rice mutant plants with reduced GA_3_-level showed a higher degree of resistance against *M. oryzae*.

## Conclusion

Elevated ABA levels function as susceptibility factor during pathogenicity of *M. oryzae* with different host plants such as barley, as shown in this study, and rice, as known from the literature. This phenomenon most likely depends on antagonistical effects disturbing balancing of the plant hormonal network. With respect to the capability of ABA in increasing resistance against powdery mildew on barley, our results with *M. oryzae* present an additional example of opposing effects of defence pathways in barley against biotrophic (powdery mildew) and hemi-biotrophic (*M. oryzae*) pathogens.

## Methods

### Fungal isolates, plants and inoculation

The spring barley (*Hordeum vulgare* L.) cultivars Ingrid, Sultan5, Golden Promise, Pallas, backcross line Ingrid*mlo5* (kindly provided by P. Schulze-Lefert, Max-Planck Institute for Plant Breeding Research, Cologne, Germany), Morex (kindly provided by P. Schweizer, IPK, Gatersleben, Germany), Steptoe and *Az34* (= *nar2a,* mutant generated in a Steptoe genetic background; both kindly provided by A. Kleinhofs, Dept. Crop and Soil Sciences, Washington State University, Pullman, USA) were used in this study. The winter barley cultivar Hannah (kindly provided by J.B. Speakman, BASF AG, Limburgerhof, Germany) was investigated additionally. Plants were cultivated in a growth chamber at 16–18°C, 50-60% relative humidity with a 16/8 h day/night cycle at 210 μmol m^−2^ s^−1^.

The *M. oryzae* isolates TH6772 (obtained from Institute of Biochemistry, Facility of Agriculture, Tamagawa University, Machida-shi, Tokyo, Japan) and BR32 (kindly provided by D. Tharreau, CIRAD, Montpellier, France) were grown on rice leaf agar (water extract from 50 g l^−1^ rice leaves, 10 g l^−1^ soluble starch, 2 g l^−1^ yeast extract (Biolabor, Muenster, Germany), 15 g l^−1^ agar). Fungal culture plates were incubated at 22°C with a 16/8 h day/night regime. For stimulation of sporulation black-light (310 to 360 nm) was added for 14 days during the illumination period. From these plates, fungal mycelium was scraped, washed off with distilled water and filtered through three layers of gauze. Conidia present in the filtrate were adjusted to a final concentration of 200,000 spores ml^−1^ in a solution containing 0.1% gelatine (v/v) and 0.05% Tween 20 (v/v). After spray inoculation with this spore solution, plants were kept in a moist chamber (26°C and nearly 100% relative humidity) for at least 22 hours and thereafter cultivated under growth chamber conditions as described above.

### Hormone application

Solutions containing plant hormones were prepared at the following concentration in distilled water supplemented with 0.01% Tween 20 (v/v): i) 0.1 mM sodium salicylate (SA), ii) 20 μM 1-aminocyclopropane-1-carboxylic acid (ACC), iii) 20 μM indole-3-acetic acid (IAA), iv) 20 μM gibberellic acid (GA_3_), v) 20 μM abscisic acid (ABA). All solutions except for SA were diluted from 4 mM methanolic stock solutions. For mock treatment a solution of Tween 20 and methanol at similar concentrations was prepared. Solutions were sprayed onto leaves of seven day old barley plants. Thereafter, the plants were incubated for one hour at growth chamber conditions and then inoculated.

### Microscopic analyses

At different timepoints after inoculation, primary leaves were detached and placed in a clearing solution (0.15% trichloracetic acid (w/v) in 4:1 ethanol:chloroform (v/v)) for at least two days and then stored in 50% glycerol until evaluation. Fungal structures were observed by bright-field microscopy using a Leica-DMBRE (Leica Microsystems, Wetzlar, Germany). Deposition of autofluorescent material was observed with epi-fluorescent light using the same microscope (excitation filter 485 nm, dichroic mirror 510 nm, barrier filter 520 nm). Images were taken with a digital camera JVC KYF 750 (JVC Professional Europe Ltd, London, UK). Progress of fungal infection and corresponding plant reactions were assessed by quantitative cytology as described previously [17,19]. Therefore, at least 100 plant-fungus interaction sites were inspected per leaf and assigned to different categories (see Figure 1). Statistical analyses were performed with SigmaStat (Systat Software Inc., San Jose, California, USA). Significance of differences among means was determined by Student’s t-test or an ANOVA with a Holm-Sidak-analysis (95% confidence) was performed.

### Phytohormone measurements by high performance liquid chromatography and mass spectrometry

Each sample for phytohormone measurements was generated by pooling five primary leaves, immediately freezing them in liquid nitrogen and storing them at −80°C. Leaf material was subsequently ground in liquid nitrogen to homogeneity and 150 mg of this powder was used for extraction. The extraction was done avoiding light exposure to exclude cis-ABA conformation changes into the biological inactive trans-ABA. Samples were extracted as described in Häffner et al. [30] and for each sample 2 ng of the deuteriated internal standard D6-(2Z,4E)-ABA (D6-ABA) (Icon Services, NJ, USA) was spiked into the extraction solution. ABA and salicylic acid (SA) were monitored by HPLC-ESI-MS/MS as described in [30] using the mass transitions of m/z 262.8 → m/z 153 (8 eV) for ABA and m/z 268.9 → m/z 159 (9 eV) for D6-ABA and m/z 136.8 → m/z 93.0 (CE 14.5 eV) for SA. Quantification of ABA was performed with a calibration curve of the ratio of peak areas of the unlabelled standard to the peak area of the deuterium-labelled standard. SA was quantified with an external calibration curve obtained with pure standard.

## Additional file

Additional file 1: Figure S1. Increase of barley susceptibility against *M. oryzae* after soil drench with ABA in different concentrations. Barley plants (cultivar Ingrid) were grown in soil for seven days and thereafter treated with either mock-solution or solutions containing different concentrations of ABA (20 mL per pot). After 48 hours plants were inoculated with *M. oryzae* isolate TH6772 (200,000 conidia mL^−1^) and seven days later disease severity was quantitatively evaluated. Therefore, typical lesions were counted per leaf and means and standard deviations were calculated from at least ten individual plants. Significant differences were determined for each treatment between ABA and mock using t-test (p ≤ 0.05) and marked with asterisks. The experiment was repeated once with similar results. **Figure S2.** Accumulation of barley *PR1b*-specific transcripts in response to ABA treatment and inoculation with *M. oryzae*. Seven day old primary leaves of barley cultivar Ingrid were sprayed either with abscisic acid solution (20 μM) or mock-solution and inoculated one hour later with *M. oryzae* isolate TH6772 (200,000 conidia mL^−1^). Five leaves were harvested per sample at time points indicated (h p.i., hours after inoculation). Total RNA was extracted and subjected to gel blot analysis as reported previously [21]. Equal loading of the gel with 10 μg of total RNA was monitored by ethidium bromide (EtBr) staining. Hybridization of the blot was done with an *in vitro* transcribed digoxigenin-labelled *PR1b*-specific probe. The experiment was repeated twice with similar results.

## Abbreviations

ABA: Abscisic acid
*Bgh*: *Blumeria graminis* f. sp. *hordei*
GA_3_: Giberellic acid
SA: Salicylic acid
