# Supplementary material for: Abscisic acid negatively interferes with basal defence of barley against Magnaporthe oryzae
Source: BMC Plant Biol. 2015 Jan 21;15:7. doi: 10.1186/s12870-014-0409-x (PMC4307682; doi:10.1186/s12870-014-0409-x)
Supplement: Additional file 1: Figure S1. — Increase of barley susceptibility against M. oryzae after soil drench with ABA in different concentrations. Barley plants (cultivar Ingrid) were grown in soil for seven days and thereafter treated with either mock-solution or solutions containing different concentrations of ABA (20 mL per pot). After 48 hours plants were inoculated with M. oryzae isolate TH6772 (200,000 conidia mL−1) and seven days later disease severity was quantitatively evaluated. Therefore, typical lesions were counted per leaf and means and standard deviations were calculated from at least ten individual plants. Significant differences were determined for each treatment between ABA and mock using t-test (p ≤ 0.05) and marked with asterisks. The experiment was repeated once with similar results. Figure S2. Accumulation of barley PR1b-specific transcripts in response to ABA treatment and inoculation with M. oryzae. Seven day old primary leaves of barley cultivar Ingrid were sprayed either with abscisic acid solution (20 μM) or mock-solution and inoculated one hour later with M. oryzae isolate TH6772 (200,000 conidia mL−1). Five leaves were harvested per sample at time points indicated (h p.i., hours after inoculation). Total RNA was extracted and subjected to gel blot analysis as reported previously [21]. Equal loading of the gel with 10 μg of total RNA was monitored by ethidium bromide (EtBr) staining. Hybridization of the blot was done with an in vitro transcribed digoxigenin-labelled PR1b-specific probe. The experiment was repeated twice with similar results. [file 12870_2014_409_MOESM1_ESM.pdf]

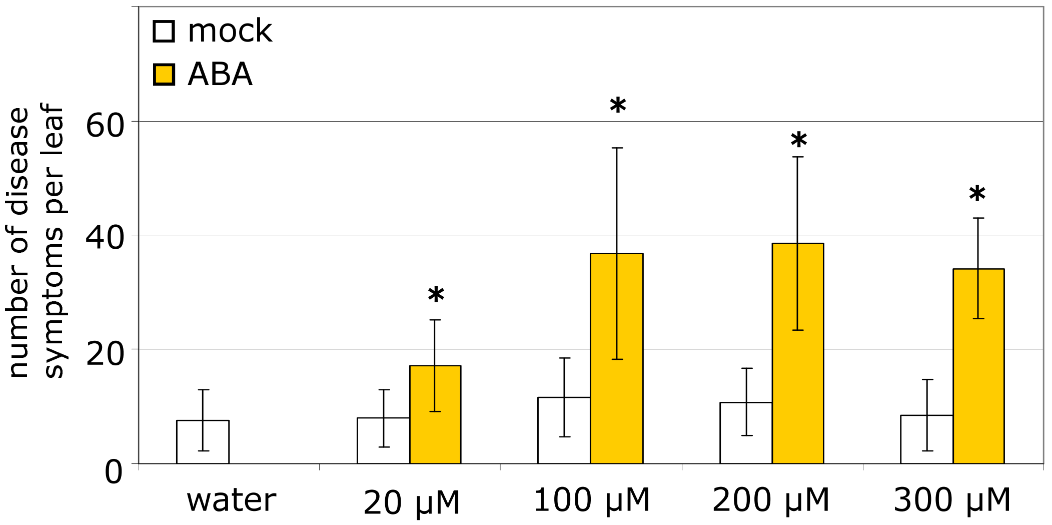

Figure S1. Increase of barley susceptibility against *M. oryzae* after soil drench with ABA in different concentrations.
Barley plants (cultivar Ingrid) were grown in soil for seven days and thereafter treated with either mock-solution or solutions containing different concentrations of ABA (20 mL per pot). After 48 hours plants were inoculated with *M. oryzae* isolate TH6772 (200,000 conidia mL^{-1}) and seven days later disease severity was quantitatively evaluated. Therefore, typical lesions were counted per leaf and means and standard deviations were calculated from at least ten individual plants. Significant differences were determined for each treatment between ABA and mock using t-test ($p \leq 0.05$) and marked with asterisks. The experiment was repeated once with similar results.

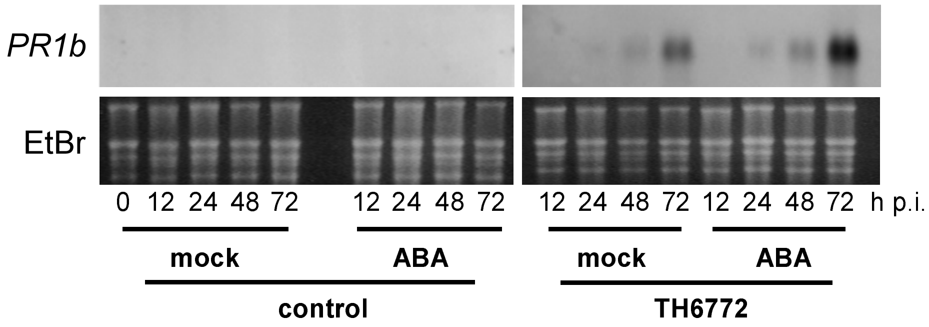

Figure S2. Accumulation of barley *PR1b*-specific transcripts in response to ABA treatment and inoculation with *M. oryzae.*
Seven day old primary leaves of barley cultivar Ingrid were sprayed either with abscisic acid solution (20 µM) or mock-solution and inoculated one hour later with *M. oryzae* isolate TH6772 (200,000 conidia mL^{-1}). Five leaves were harvested per sample at time points indicated (h p.i., hours after inoculation). Total RNA was extracted and subjected to gel blot analysis as reported previously [21]. Equal loading of the gel with 10 µg of total RNA was monitored by ethidium bromide (EtBr) staining. Hybridization of the blot was done with an *in vitro* transcribed digoxigenin-labelled *PR1b*-specific probe. The experiment was repeated twice with similar results.
